# Insights into the Effect of Curcumin and (–)-Epigallocatechin-3-Gallate on the Aggregation of Aβ(1–40) Monomers by Means of Molecular Dynamics

**DOI:** 10.3390/ijms21155462

**Published:** 2020-07-30

**Authors:** Francesco Tavanti, Alfonso Pedone, Maria Cristina Menziani

**Affiliations:** 1 CNR–NANO Research Center S3, Via Campi 213/a, 41125 Modena, Italy; 2 Department of Chemical and Geological Sciences, University of Modena and Reggio Emilia, Via Campi 103, 41125 Modena, Italy; alfonso.pedone@unimore.it (A.P.); mariacristina.menziani@unimore.it (M.C.M.)

**Keywords:** computational simulation, amyloid, Alzheimer, curcumin, EGCG

## Abstract

In this study, we compared the effects of two well-known natural compounds on the early step of the fibrillation process of amyloid-β (1–40), responsible for the formation of plaques in the brains of patients affected by Alzheimer’s disease (AD). The use of extensive replica exchange simulations up to the µs scale allowed us to characterize the inhibition activity of (–)-epigallocatechin-3-gallate (EGCG) and curcumin (CUR) on unfolded amyloid fibrils. A reduced number of β-strands, characteristic of amyloid fibrils, and an increased distance between the amino acids that are responsible for the intra- and interprotein aggregations are observed. The central core region of the amyloid-β (Aβ(1–40)) fibril is found to have a high affinity to EGCG and CUR due to the presence of hydrophobic residues. Lastly, the free binding energy computed using the Poisson Boltzmann Surface Ares suggests that EGCG is more likely to bind to unfolded Aβ(1–40) fibrils and that this molecule can be a good candidate to develop new and more effective congeners to treat AD.

## 1. Introduction

The presence of senile plaques made by amyloid-β (Aβ) fibrils in the brain is one of the hallmarks of the Alzheimer’s disease [1,2,3]. Aβ fibrils are composed by monomers of 40–42 amino acids that, under normal conditions, have an unfolded and disordered structure without toxic effects. However, under pathological conditions, the Aβ monomers self-associate to form larger aggregates such as oligomers and fibrils. The self-aggregation process is composed by several steps, of which the first step is the primary nucleation. At the beginning, the disordered monomers fold themselves into nuclei, i.e., aggregates where the monomer addiction is faster than its dissociation with a consequent growth in size and length, which is called elongation [4]. However, these oligomers are temporary, and they undergo to other aggregation steps that result in larger and longer fibrils. One of the most promising way to treat Alzheimer’s is to inhibit the first aggregation step using natural compounds [5,6]. The aggregation pathway of aberrant proteins and the mechanism to inhibit the aggregation are of fundamental importance to the development of more effective drugs [7].

One of the promising approaches is to inhibit the amyloid folding before the formation of β-sheet-rich aggregates characteristic of mature folded amyloids.

In the last years, epidemiological studies on the effects of the diet against AD and dementia suggested that the high intake of flavonoids and polyphenols found in fruits and vegetables [8], such as in the Mediterranean diet, reduces the risk of AD and cognitive impairments [9,10]. Several natural molecules have been identified to promote cognitive health and to interfere with the amyloidogenic activity in AD [11,12].

A detailed knowledge of how natural compounds interact with Aβ fibrils is a prerequisite for the rationalization of their antiaggregating activity. Unfortunately, despite intensive research, the experimental characterization remains a great challenge.

Computer simulations have been extensively used to provide atomistic-level information on the molecular mechanisms of the interaction of the amyloid oligomer with ligands [7,13,14].

In this study, the effect of some natural compounds on the early step of Aβ(1–40) fibril nucleation will be studied at the atomic level by means of molecular simulations. Two widely studied molecules with known anti-amyloidogenic activity have been chosen. Curcumin (CUR) is a well-known bio phenol, found in the rhizome of turmeric (*Curcuma longa*), with anti-inflammatory and antioxidants activity, and recently, it has been demonstrated that it is able to bind to small amyloid-protofibrils and to block the aggregation and formation of fibrils in vitro and in vivo [5,8]. (–)-epigallocatechin-3-gallate (EGCG) is a flavonoid, found in green tea (*Camellia sinensis*), with the inhibition of α-synuclein and Aβ fibrillogenesis [15,16]. At the best of our knowledge, this is the first study on the interaction of the full-length unfolded Aβ(1–40) fibril in interactions with CUR and EGCG at different fibril/ligand ratios performed by extensive replica exchange solute tempering molecular dynamics simulations in an explicit solvent.

## 2. Results

### 2.1. Structural Analysis of Monomers in the Solution

#### 2.1.1. Secondary Structures

Starting from the unstructured Aβ(1–40) monomeric unit, the time evolution of the secondary structures for monomeric, dimeric and trimeric peptides has been followed during the interval 100–800 ns of replica exchange solute tempering (REST) simulations. In general, the formation of α-helix and β-strands localized in different regions of the Aβ monomers can be observed after 10–20 ns (see Figure S1).

A more detailed analysis is provided by the secondary structure propensity for each amino acid of the Aβ monomers in water solution and has been computed for the Aβ monomeric, dimeric or trimeric models.

#### 2.1.2. Aβ Monomeric Model

**Figure 1:**
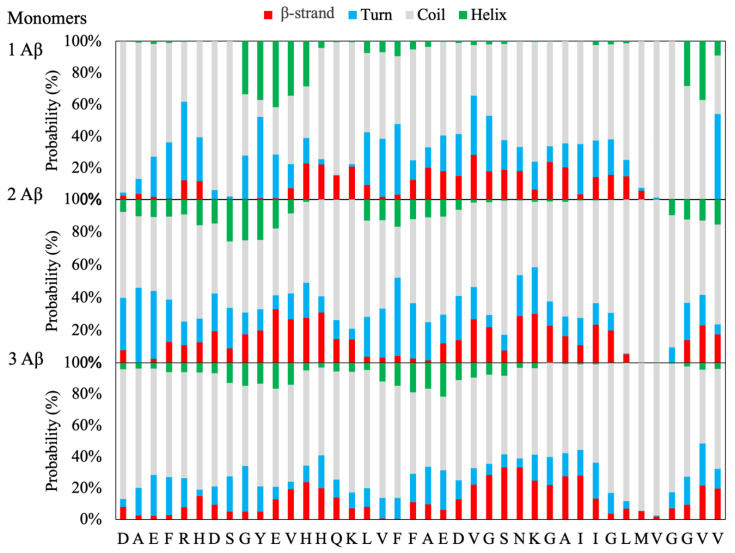
Secondary structure propensity for the amyloid-β (Aβ) monomers in the solution.

The results of the REST simulation for the Aβ monomeric model (Figure 1) show a preference for the ^9^GYEVHH^14^ stretch of amino acids to fold in a helix structure (helical propensity around 40%) in substantial agreement with the NMR structure of the Aβ-(1–42) peptide in a water solution in which the helix spans the whole stretch from Tyr10 to Asp22 [17]. In addition, a moderate strand propensity of around 20% for the single Aβ monomer is observed for the stretch of amino acids ^13^HHQK^16^ and D22–L34. A central role as a promoter of β conformational transition has been recently recognized for the 25–35 sequence, as a function of the polarity of the environment [18]. It is worth recalling that mature Aβ fibrils have a common structure characterized by a β-sheet formed by two antiparallel β-strands located at residues ^15^QKLVFFA^21^ and ^30^AIIGLMVGGV^39^, while the amino acids close to the N-terminal are unfolded [19,20,21,22] (see the Methods section). Therefore, in view of the observed secondary structure propensity, the ^13^HHQK^16^ and D22–L34 stretch of amino acids may be considered to constitute peptide regions that seed the conformational transitions to the characteristic β-folding.

#### 2.1.3. Aβ Dimeric and Trimeric Models

Interestingly, by increasing the number of peptides in the simulation box, a significant increase of the β-strands propensity, both in terms of the number of amino acids and probability, is observed at the expenses of the helical propensity, which decreases dramatically for the dimeric model and almost disappears in the trimeric model (Figure 1). Moreover, a shift of the two putative β-strands towards the N-terminal (^9^GYEVHHQ^15^) and C-terminals (^24^VGSNKGAII^32^) is observed. This behavior supports the hypothesis that the increased number of monomeric peptides in solution results in the formation of β-rich structures similar to that of the mature fibril [22,23].

#### 2.1.4. Aβ Monomeric, Dimeric and Trimeric Models with Ligands

**Figure 2:**
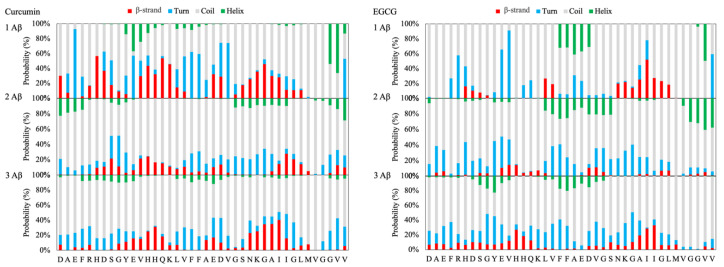
Secondary structure propensity for Aβ monomers interacting with curcumin (CUR) (left) and (–)-epigallocatechin-3-gallate (EGCG) (right).

The insertion of CUR in the simulation box has a significative influence on the folding of the Aβ peptides. In fact, a substantial decrease in the propensity of the monomeric peptide to adopt an α-helix conformation is observed (see Figure S2). Moreover, the increasing of the number of monomers and ligands in the simulation boxes results in a dramatic reduction in the propensity of both strands and helices in favor of random coil, as shown in Figure 2 and in the Supplementary Information.

In the case of EGCG, the strand’s propensity is consistently lowered and is observed almost only in the region ^27^KGAIIGLM^35^ for the monomeric model, while there is a slight tendency to form a helix-turn in the region ^19^FFAED^23^ (see Figure 2 and Figure S3), which, in mature fibrils, corresponds to the end of the β-1 β-strand and the beginning of the turn connecting to the β-1 and β-2 β-strands. These findings agree with the results of a structural characterization of EGCG-induced Aβ oligomers employing solution states and magic angle spinning (MAS) solid-state NMR techniques, which showed that the C-terminal part of the Aβ peptide (residues 22–39) adopts a β-sheet conformation, whereas the N-terminus (residues 1–20) is unstructured [24].

In summary, the results support the hypothesis that both CUR and EGCG reduce the formation of the β-strand structure’s characteristics of mature Aβ(1–40) fibrils (see Figures S4–S9).

#### 2.1.5. Radius of Gyration

The radius of gyration (R_g_) for Aβ monomers in a solution gives an estimation of the compactness of the protein. According to Kolinski and Skolnick [25], the mean squared radius of gyration <*Rg*> for single domain globular proteins is given by: <*Rg*> = 0.22*n*^0.38^, where n is the number of residues. Therefore, for a globular protein of 40 amino acid residues, a Rg of 0.89 nm should be expected. Higher values correspond to more extended structures.

**Figure 3:**
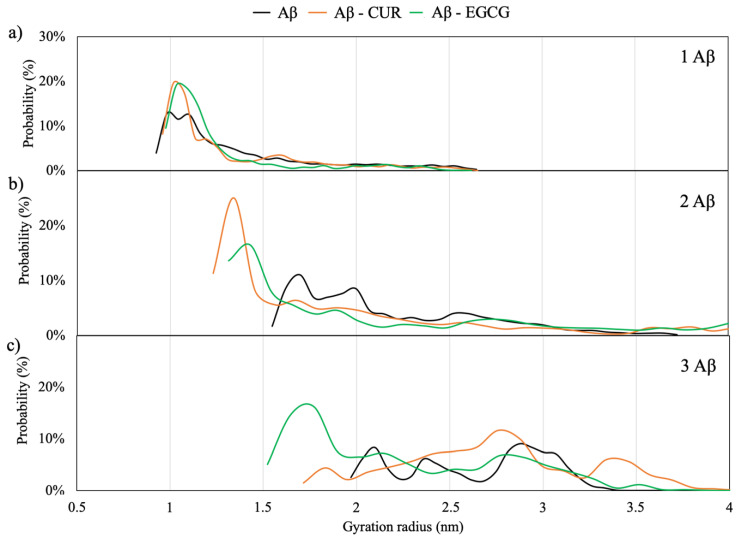
Distribution of the gyration radius (in nm) for the Aβ monomers, with and without ligands. Panel (**a**) for the single monomer, (**b**) for two monomers and (**c**) for three monomers.

Figure 3 shows the probability distribution of R_g_ for the Aβ monomers in the solution and the effects of EGCG and CUR additions on the distribution. It can be observed that the single Aβ monomer adopts a radius of gyration from 0.9–1.2 nm characteristic of a collapsed, almost globular, protein. This value well agrees with single-molecule-level fluorescence values for monomeric Aβ42 (Rg 0.9 ± 0.1 nm) peptides [26]. The almost spherical aggregation of the monomers is enhanced by the presence of the two ligands.

By increasing the number of Aβ monomers, the maximum in the R_g_ distribution is shifted to higher values, corresponding to a more extended structure, of 1.7 nm for the dimeric model and 2–3 nm for the trimeric model, in agreement with the values of 1.5–2.3 nm found for small oligomers by means of a fluorescence correlation spectroscopy of wild-type and mutated Aβ40 peptides [27]. The elongation of the structures is in-line with the first step of the self-aggregation process of the Aβ monomers, called primary nucleation, proposed by Arosio et al. [4]. Interestingly, the insertion of CUR and EGCG in the simulation box of the dimeric and trimeric peptides shifts the maximum in the R_g_ distribution to lower values corresponding to about 1.4 nm and 1.7 nm, respectively. The ability of EGCG to convert Aβ fibrils into small spherical oligomers has been also demonstrated by means of NMR [16,26] and combined electron microscopy, circular dichroism and thioflavin T-binding assay studies [15]. Indeed, the profile of the R_g_ distribution for the CUR and EGCG-trimeric Aβ peptide indicates that CUR has a smaller influence on the monomer’s elongation with respect to EGCG.

### 2.2. Intra-and Inter-Monomer Contacts

#### 2.2.1. Intra-Monomer Contacts

Specific interactions between amino acids of the Aβ (1–40) and Aβ (1–42) monomers have been identified by means of low-resolution solid–state NMR, site-specific mutagenesis studies, electron paramagnetic resonance data, tip-enhanced Raman spectroscopy and other experimental techniques. These interactions are achieved during the intermediate steps of monomer folding and are considered to act as seminal internal constraints for amyloid formation [13,28].

In particular, the salt bridge between the amino acid residues D23 and K28 stabilizes the turn that builds the cross-β structure [21,28,29], and the hydrophobic contact between F19 and L34 are considered to constitute the first interaction between the two hydrophobic stretches of amino acids that build the β structure in mature fibrils [30,31,32].

Therefore, though the Aβ peptides adopt highly heterogeneous ensembles, monitoring the trajectories of these couple of amino acids during dynamics enables considerations on the correct folding process to be done.

**Figure 4:**
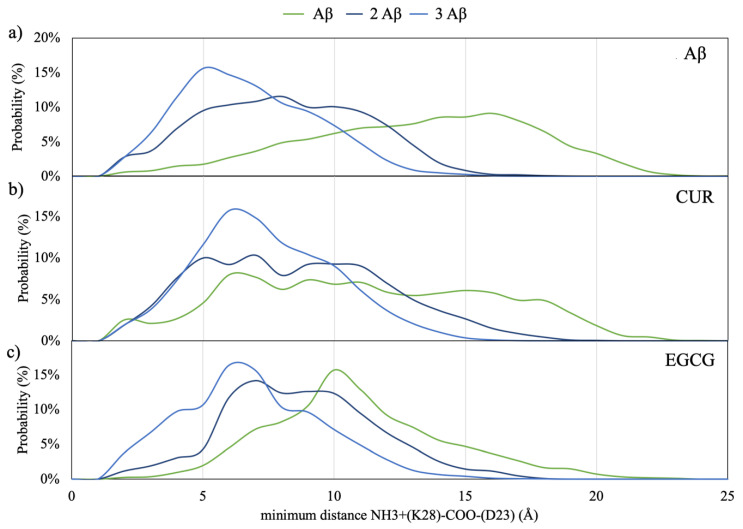
Distribution of the minimum distances between the NH_3_^+^ of K28 and the COO^–^ of D23. The minimum distance is 2.8± 0.2Å, while the average distance is 3.7 ± 0.3Å [14]. Panel (**a**) for the monomers, (**b**) for monomers with CUR and (**c**) for monomers with EGCG.

The distribution of the minimum distances between D23 and K28 in Aβ monomers in the solution is reported in Figure 4. By taking into account that structural constraints from solid-state NMR and electron microscopy suggest an average distance between the NH_3_^+^ of K28 and the COO^–^ of D23 of 3.7Å in the folded Aβ peptides [22] but electrostatic interactions between charged residues are still significant at longer distances, a substantial increased propensity to form this salt bridge is manifested by the dimeric and trimeric Aβ models with respect to the monomeric (Figure 4a).

The insertion of CUR and EGCG in the simulations box (Figure 4b,c) does not perturb this behavior but slightly shifts the maximum of the distance probability to higher distances.

**Figure 5:**
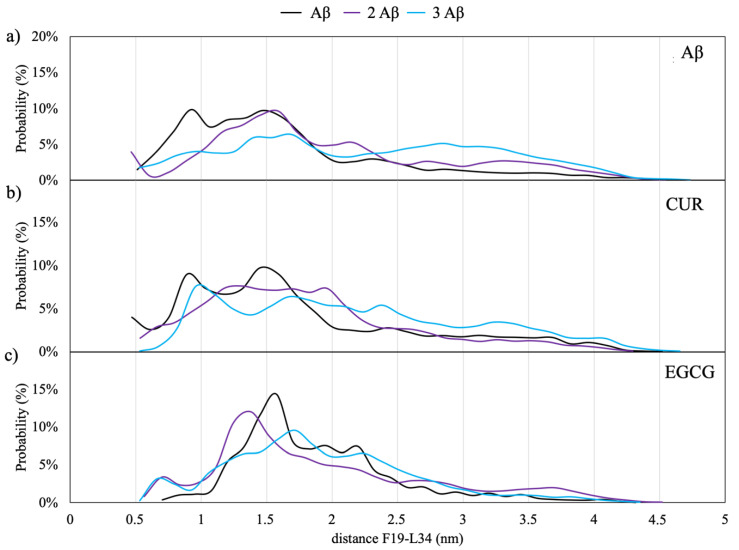
Distribution of the distances between the Cα of F19 and L34. Panel (**a**) for the monomers, (**b**) for monomers with CUR and (**c**) for monomers with EGCG.

Figure 5a shows the distribution of minimum distances between F19 and L34; the average distance value between the Cα of F19 and L34 found in mature fibrils by means of solid-state NMR is 9.5 ± 1 Å [20,22,23].

The results of the REST simulations of Aβ monomers in the solution seem to indicate that a close contact between the two amino acids is more probable for the monomeric model; in this case, there is a 10% of probability of occurrence of a distance of 9 Å, which allows the establishment of Van der Waals interactions between the side chains of F19 and L34.

A similar behavior is observed for the models with CUR, as shown in Figure 5b. On the contrary, in the case of EGCG (Figure 5c), the maximum in the distance distributions are shifted to higher values between 12 and 20 Å, indicating that this molecule is able to inhibit the formation of the hydrophobic contacts between F19 and L34. This finding agrees with a very recent saturation transfer difference NMR study [33], which showed that EGCG dramatically remodels the contacts between Ab monomers and the oligomer surface.

#### 2.2.2. Inter-Monomer Contacts

Several topologies of the inter-protofilament interface in Aβ40 fibrils have emerged by solid-state NMR experiments, differences being dictated by experimental conditions.

The organization of Aβ monomers into agglomerates composed by two (two-fold topology) [20,22,34,35] or three (three-fold topology) [36] protofilaments in different arrangements have been obtained.

Contacts that provide direct constraints on the quaternary structure of the protofibrils and are shared by all the arrangements have been identified experimentally as established mainly by the side chains of M35 [22,35,37] and one or more of the residues I31, I32 and M35 of one monomer/protofilament with one or more of residue G37, G39 and V29 of a second one [20,22,35,36].

**Figure 6:**
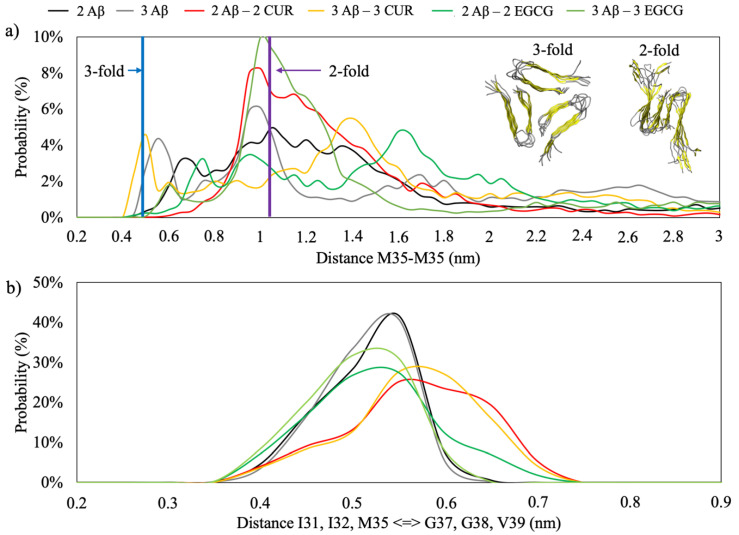
In panel (**a**), the distribution of distances between the M35–M35 Cα in the REMD simulations. The purple and blue bars represent the average distance of the folded Aβ(1–40) fibrils in the 2-fold and 3-fold configurations, respectively. In panel (**b**), the distribution of the minimum distance between the Cα of I31, I32 and M35 and G37, G38 and V39.

The MD simulation of the two-fold topology of the Aβ(1–40) fibril with the β2 β-sheets facing each other shows an inter-monomeric distance between the Cα of M35 in the order of 1.1 nm, while, in the three-fold topology, the distance is reduced to 0.5 nm. Figure 6a shows the distribution of the M35-M35 distances in the REMD simulations. A marked trend to assume a configuration similar to the two-fold topology is observed. Moreover, the profile of the minimum distance between the Cα of amino acids I31, I33 and M35 versus G37, G38 and V39 (Figure 6b) has a distribution centred at 5.5Å, which is consistent with the shortest distance between I31 of G37 of 5.2Å found by NMR experiments [22].

These results indicate that the first step of fibril recognition necessary for primary nucleation is reached during the simulation time.

It is interesting to note that in the simulation CUR acts as an inhibitor of the aggregation; in fact, the presence of two or three molecules of CUR shifts the distance distribution towards higher values (Figure 6b).

### 2.3. Ligand–Aβ Monomer Interactions

The estimation of the binding free energy of the ligand-Aβ40 oligomer interactions provides quantitative information on the binding determinants.

Table 1 shows the binding free energy Δ$G_{\mathrm{binding}}$ obtained using the molecular mechanic-Poisson-Boltzmann surface area (MM-PBSA) method. The analysis of the energy components clearly indicates that the VdW term is the principal actor in the interaction of both EGCG and CUR, whereas the nonpolar term, Δ$G_{\mathrm{surf}}$, furnishes a negligible contribution. Moreover, in all cases, the EGCG binds stronger than CUR to Aβ, with an average value of 30 kcal/mol, while the strength of the CUR-Aβ interaction decreases when dimeric and trimeric models are considered going from –25 kcal/mol for the monomer to –18 kca/mol for the trimer.

**Figure 7:**
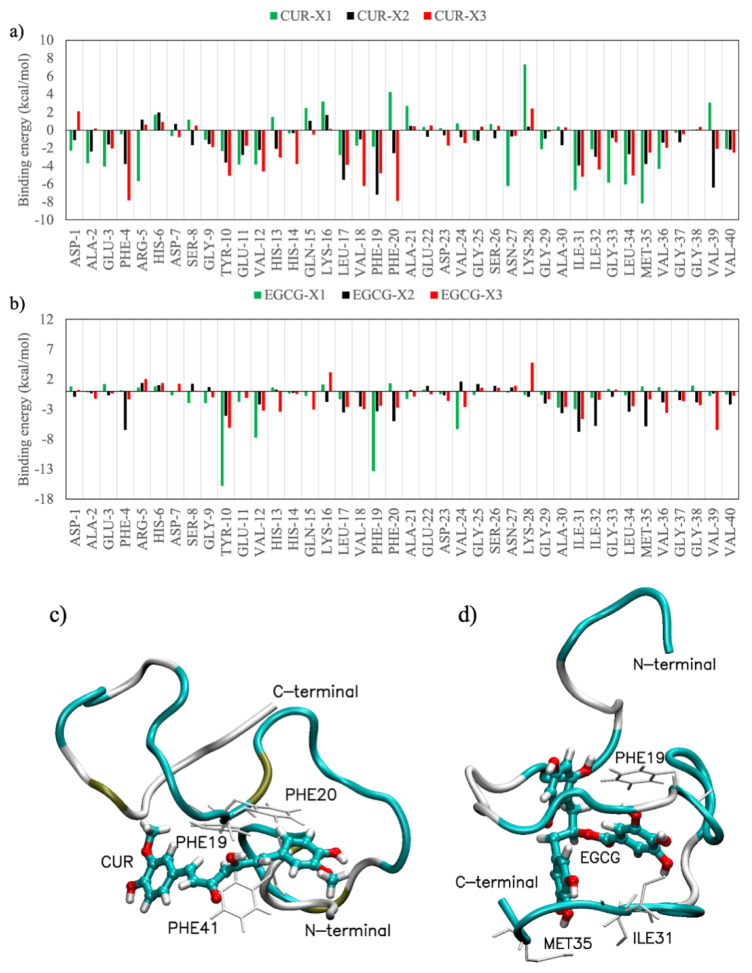
Average contribution of each residue of the Aβ(1–40) to $\Delta G_{\mathrm{binding}}$ with CUR (panel **a**) and EGCG (panel **b**) mediated over all simulations. Graphical representation of CUR (panel **c**) and EGCG (panel **d**) interacting with hydrophobic amino acids labeled with their names and numbers.

Figure 7 shows the contribution of each residue to the binding energy with ligands. The data values are mediated over all the computational simulations obtained by the MM-PBSA analysis. It can be noted that both CUR and EGCG interact mainly with hydrophobic amino acids such as F4, Y10, V12, L17, V18, F19, F20, I31, I32, L34, M35, V36 and V39 [38], where in bold are highlighted the amino acids that strongly interact both with CUR and with EGCG. The 2M9R PDB structure is a complex between the amyloid-beta peptide (1–40) and the polyphenol epsilon-viniferin glucoside ligand. This ligand is similar in the size and number of π-rings to EGCG and, in agreement with our finding, gives rise to a coulombic interaction with the amine group of Q15 and to the π-π interaction with F19. Overall, the results confirm the nonspecificity of the CUR and EGCG binding previously observed by both experimental [24,33,39] and computational studies [40].

## 3. Discussion

In this work, extensive replica exchange solute tempering molecular dynamic simulations of Aβ(1–40) monomers, dimers and trimers interacting with natural polyphenols curcumin and (–)-epigallocatechin-3-gallate have been performed. The results clearly indicate that the tendency to form β-sheet-rich structures, characteristic of amyloid fibrils, in dimeric and trimeric models is reduced by the addition of both ligands, whereas an increase of turn and helical structures is observed. However, CUR has a smaller influence on the perturbation of monomer elongations with respect to EGCG, as also detected by the results obtained by monitoring the radius of the gyration profiles and the distributions of the F19-L34 distances, which can be considered first marks of the interaction between the two hydrophobic regions of the Aβ(1–40) peptide. The central hydrophobic core of Aβ, which is responsible for the inter- and intra-molecular contacts in folded fibrils, is found to provide important interacting sites for both ligands, with Van der Waals interactions being the main driving forces for ligand binding.

Overall, the results indicate that EGCG and CUR have two different inhibition mechanisms, but EGCG seems to be the most efficient of the two natural compounds in preventing early nucleation, precluding toxic effects associated with the fibril growth process. This study paves the way for further investigations of other potential effective natural compounds to inhibit the amyloid-β aggregation and to prevent Alzheimer’s disease.

## 4. Materials and Methods

### 4.1. Molecular Dynamics Simulations

The GROMOS 54a7 force field [41] was used to perform the molecular dynamics simulations, since it has been shown to stabilize the secondary structure elements in close agreement with the experimental observations [42]. The protonation state of the ligands at pH 7 was considered, and the relative force field parameters in the Gromacs format [43,44] were assigned using the Automated Topology Builder [45,46] web server, as previously done [14,46].

**Figure 8:**
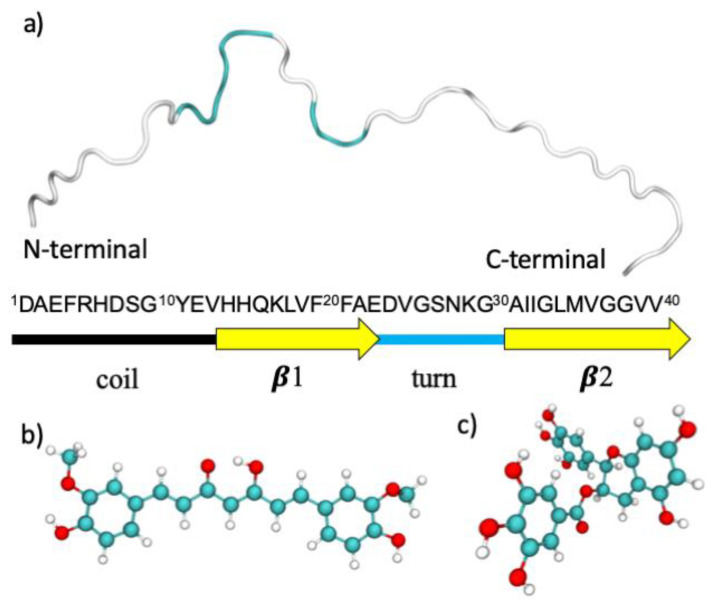
(**a**) The Aβ (1–40) monomeric unit obtained from the 2M9R PDB structure colored accordingly to its secondary structure (random coil is represented in white and turns in light blue). The amino acid sequence is also reported with the secondary structure assignment of the folded Aβ (1–40). Structural representation of (**b**) the curcumin molecule in its keto–enolic form (CUR) and (**c**) (–)-epigallocatechin-3-gallate (EGCG).

The structural model of the amyloid monomer in its unfolded state was retrieved from the Protein Data Bank [47]. The NMR structure of a complex between the amyloid-beta peptide (1–40) and the polyphenol epsilon-viniferin glucoside was retrieved from the PDB (PDB ID: 2M9R [48]). A representative structure of the unfolded state was selected, as shown in Figure 8, and, after removing the ligands, standard protonation states corresponding to pH 7 were assigned to ionizable amino acids. The tautomeric state of the histidine residues in the Aβ42 peptide is (εεε). The role of the tautomeric state of the histidine residues in the aggregation properties of the Aβ40 and of the Aβ42 monomers was studied by Brännström et al. [49] and by Lee and coworkers [50,51], respectively. Their results show a preference of the (εεε) isomer of the H6-H13-H14 residues to trigger β-sheet-folding.

The side of the simulation box (8.175 × 8.175 × 8.175 nm) was chosen in order to include the full-length monomer in its unfolded state. For each model (Aβ-amyloid with no ligands, Aβ-amyloid with curcumin molecule, CUR and Aβ-amyloid with (–)-epigallocatechin-3-gallate (EGCG)), three simulation boxes were built containing one (i.e., BOX_1), two (i.e., BOX_2) and three (i.e., BOX_3) monomers, respectively. In each simulation box, the ratio of Aβ-amyloid peptide:ligand is 1:1, as shown in Table 2.

Each box contains about 17,000 simple point charge water molecules [52]. Counter ions (Na^+^ and Cl^−^) were added at random locations to neutralize the systems, considering an ion concentration of 150 mM, close to the physiological value.

Each system was first minimized, until the maximum force applied to each atom was smaller than 1 kJ/(mol nm). Then, the system was equilibrated for 2 ns in the NVT ensemble where the temperature was controlled using a velocity-rescaling thermostat with a coupling time of 0.1 ps. Then an NPT equilibration of 2 ns was performed using the Berendsen barostat. Lastly, the production run was performed using the Parrinello–Rhaman barostat with a coupling time of 2 ps and an isothermal compressibility of 4.5 × 10^−5^ bar^−1^ with a timestep of 2.0 fs. The particle-mesh Ewald algorithm was used to calculate long-range electrostatics [53], with a fourth-order cubic interpolation, a grid spacing of 0.16 nm and a real space cut-off of 1 nm [54]. Both Van der Waals and neighbor list cut-offs describing short-range interactions were set to 1.0 nm. Data analysis was performed using the Gromacs package [44].

### 4.2. REST Simulations

Replica exchange solute tempering (REST) [55] was used in order to efficiently sample the protein configurations of the monomeric, dimeric and trimeric models of the Aβ peptide with and without the ligands.

In REST simulations, several replicas of the system are built by setting different temperatures to the solute, keeping the solvent at a constant temperature. REST simulations follow the same rules of standard REMD simulations, where, in each given timestep, the replicas are exchanged in temperatures if they satisfy the detailed balance condition, which preserves the Boltzmann distribution at each temperature. REST has the advantage, with respect to REMD, to employ less replicas (four to five times less) without compromising the temperature range or the efficiency of the random walk [40,46].

Eight replicas with temperatures ranging from 300 K to 440 K by steps of 20 K were simulated; replica exchanges were attempted each 2 ps, and the acceptance ratio of 20% was chosen [14,16]. Each replica in the REST simulations is 100-ns-long, for a total of 800 ns for each system and for a total simulation time of 2.4 µs; replicas were exchanged each 500 steps, corresponding to 1 ps [14,16].

### 4.3. MM-PBSA

The molecular mechanic-Poisson-Boltzmann surface area (MM-PBSA) method calculates the binding free energy of a ligand with a protein [56]. The last 10ns of each simulation were used to perform the calculation, in order to have a stable binding of Aβ with the ligand.

The binding free energy (ΔG_binding_) is the difference between the free energy of the complex, G_complex_, and the summation of the free energy of the protein, G_protein_, and ligand, G_ligand_:

(1) $$\Delta G_{\mathrm{binding}}=G_{\mathrm{complex}}-G_{\mathrm{protein}}-G_{\mathrm{ligand}}$$

Each term of the free energy is defined as:

(2) $$G=E_{\mathrm{MM}}+G_{\mathrm{solvation}}-T\Delta S$$

where the mechanical energy, E_MM_, of the solute in the gas phase is given by the summation of the bonds, angles, dihedrals, Van der Waals and electrostatic terms:

(3) $$E_{\mathrm{MM}}=E_{\mathrm{bond}}+E_{\mathrm{angle}}+E_{\mathrm{dihedral}}+E_{\mathrm{electr}}+E_{\mathrm{VdW}}$$

The solvation energy, G_solvation_, is described by the sum of nonpolar and electrostatic contributions:

(4) $$G_{\mathrm{solvation}}=G_{\mathrm{surf}}+G_{\mathrm{PB}}$$

while T and S represent the temperature and entropy, respectively. The entropic term, TΔS, is computed using the Quasi-harmonic formula [57].

The nonpolar solvation term, G_surf_, was approximated on the solvent-accessible surface area (SASA) derived from the Shrake–Rupley numerical method [58]:

(5) $$G_{\mathrm{surf}}=\gamma \mathrm{SASA}+\beta$$

with γ = 0.0072 kcal/mol Å^2^ and β = 0 [59,60].

The term comprising the electrostatic potential between the solute and the solvent, G_PB_, is calculated using the continuum solvent approximation [61] by the APBS package [56].

### 4.4. Secondary Structure Calculation

The content of secondary structures for Aβ has been computed using the DSSP algorithm [62] by performing the average on the overall simulation time for each replica.

## Figures and Tables

**Table 1:** Molecular mechanic-Poisson-Boltzmann surface area (MM-PBSA) binding free energy (Δ$G_{\mathrm{binding}}$) in kcal/mol of curcumin (CUR) and (–)-epigallocatechin-3-gallate (EGCG) for monomeric, dimeric and trimeric models. For each model, the average value for the interaction between one molecule of the ligand and one amyloid-β (Aβ) peptide is reported.

	Models	$\Delta {G_{\mathrm{Vdw}}}_{\;}$	$\Delta {G_{\mathrm{elec}}}_{\;}$	$\Delta {G_{\mathrm{PB}}}_{\;}$	$\Delta {G_{\mathrm{surf}}}_{\;}$	TΔS	$\Delta G_{\mathrm{binding}}$
A-β–EGCG	1:1	−33.01 ± 10.46	−6.45 ± 3.03	22.24 ± 7.33	−3.74 ± 1.05	12.51 ± 0.4	−33.52
	2:2	−33.44 ± 13.67	−5.92 ± 3.89	22.14 ± 9.75	−3.71 ± 1.45	7.02 ± 1.11	−27.97
	3:3	−71.62 ± 16.21	−6.97 ± 5.37	24.37 ± 13.82	−4.10 ± 1.40	5.03 ± 2.00	−30.04
A–β–CUR	1:1	−25.61 ± 6.23	−7.51 ± 4.73	23.87 ± 8.14	−3.25 ± 0.72	12.72 ± 0.83	−25.22
	2:2	−26.95 ± 10.90	−8.31 ± 6.60	25.60 ± 14.01	−3.29 ± 1.42	7.11 ± 1.07	−20.07
	3:3	−25.31 ± 13.67	−7.99 ± 8.66	23.47 ± 16.54	−3.26 ± 1.32	5.14 ± 2.37	−18.23

**Table 2:** Details of the model system set-up.

Molecules	No Ligands		CUR	EGCG
**BOX_1**	Composition	1 monomer	1 monomer, 1 CUR	1 monomer, 1 EGCG
	Number of water molecules	17,624	17,603	17,601
	Number of ions (Na^+;^ Cl^–^)	52; 49	52; 49	52; 49
**BOX_2**	Composition	2 monomers	2 monomers, 2 CUR	2 monomers, 2 EGCG
	Number of water molecules	17,456	17,414	17,412
	Number of ions (Na^+;^ Cl^–^)	55; 49	55; 49	55; 49
**BOX_3**	Composition	3 monomers	3 monomers, 3 CUR	3 monomers, 3 EGCG
	Number of water molecules	17,270	17,218	17,215
	Number of ions (Na^+;^ Cl^–^)	58; 49	58; 49	58; 49

## Supplementary Materials

Supplementary materials can be found at https://www.mdpi.com/1422-0067/21/15/5462/s1. Secondary structure assignments and the propensity for each simulation using the DSSP algorithm.
