# Supplementary material for: Insights into the Effect of Curcumin and (–)-Epigallocatechin-3-Gallate on the Aggregation of Aβ(1–40) Monomers by Means of Molecular Dynamics
Source: Int J Mol Sci. 2020 Jul 30;21(15):5462. doi: 10.3390/ijms21155462 (PMC7432714; doi:10.3390/ijms21155462)
Supplement: Supplementary file 1 [file ijms-21-05462-s001.pdf]

Supporting Materials

Insights into the effect of Curcumin and (-)-epigallocatechin-3-gallate on the aggregation of Aβ(1-40) monomers by means of molecular dynamics

Francesco Tavanti[a,b,*], Alfonso Pedone[b] and Maria Cristina Menziani[b]

[a] CNR-NANO Research Center S3, Via Campi 213/a, 41125 Modena, Italy

[b] Department of Chemical and Geological Sciences, University of Modena and Reggio Emilia, Via Campi 103, 41125, Modena, Italy

* Correspondence: *To whom correspondence should be addressed.E-mail: francesco.tavanti@nano.cnr.it

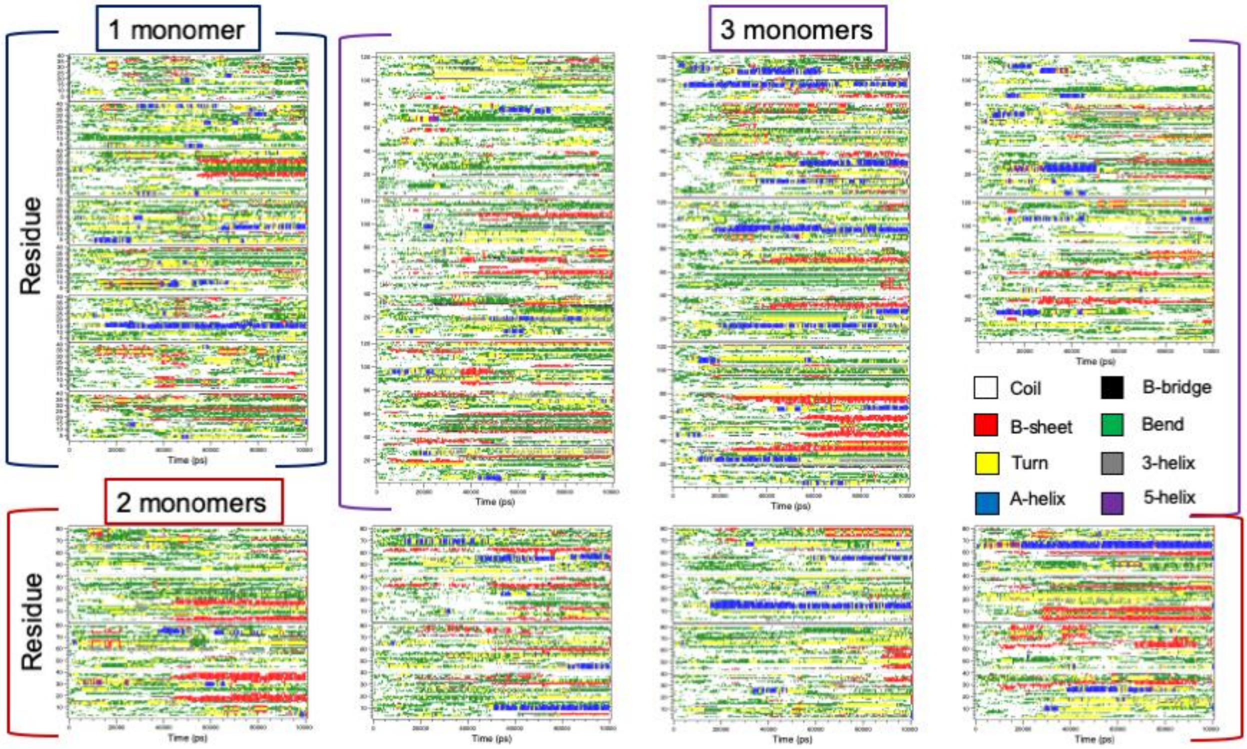

Figure S 1: Time evolution of the secondary structure for each residue of the Aβ-amyloid monomeric, dimeric, and trimeric models obtained using the DSSP software for the eight REST simulations. Each color represents a different secondary structure as explained in the legend on the right.

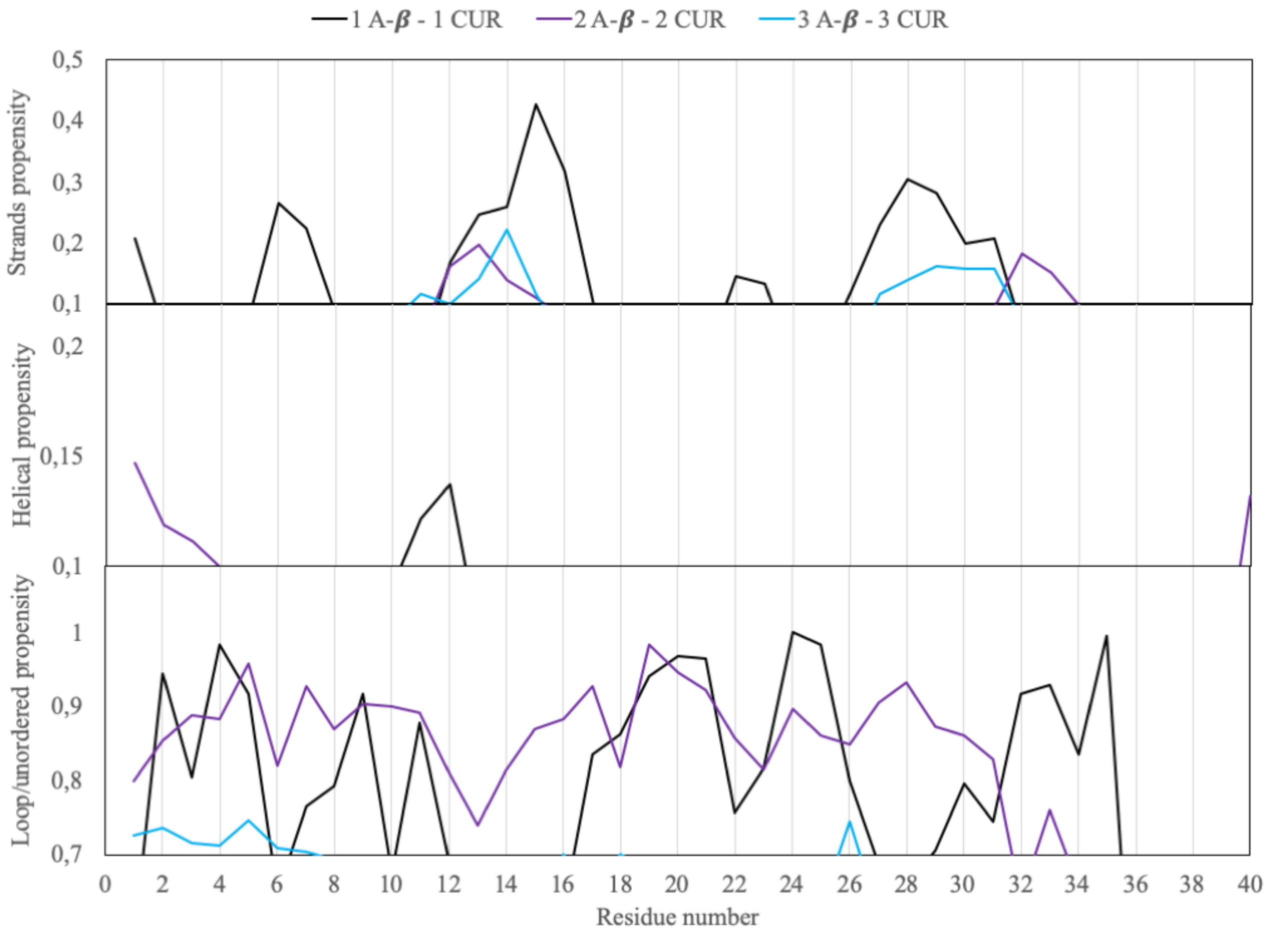

Figure S 2: secondary structure propensity for Aβ monomers interacting with CUR.

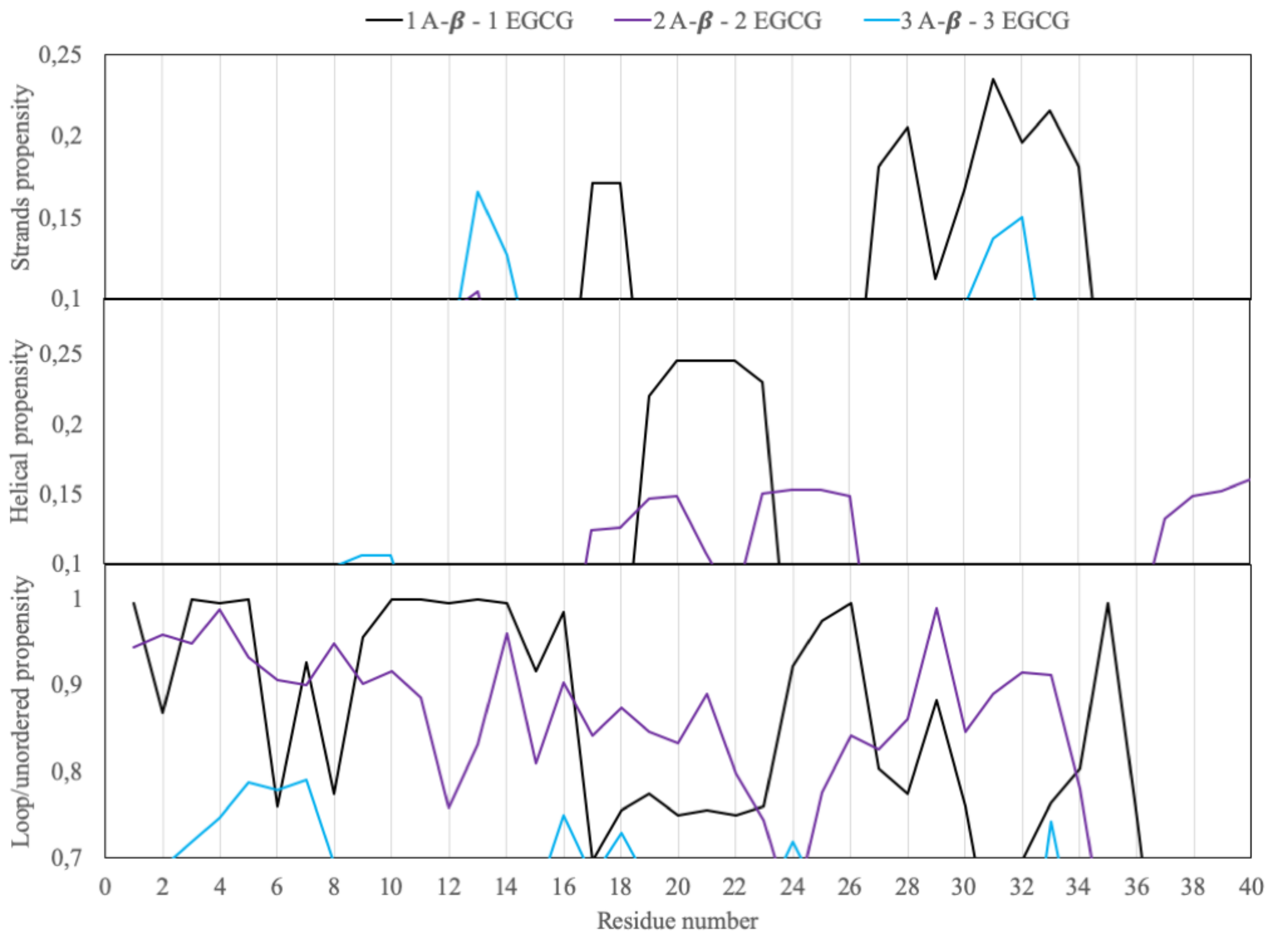

Figure S 3: secondary structure propensity for Aβ monomers interacting with EGCG.

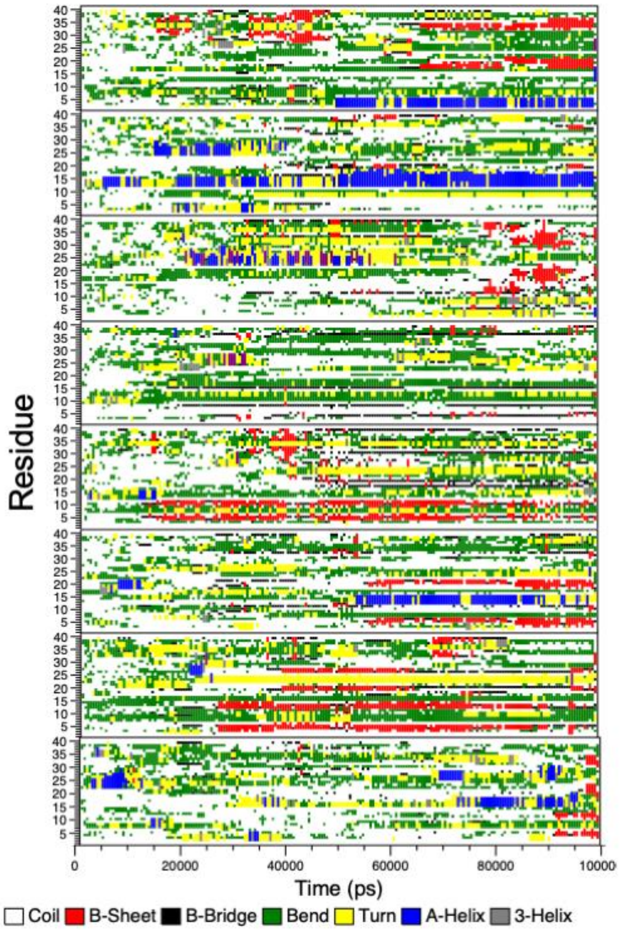

Figure S 4: Secondary structure assignment of the single monomer for each residue obtained using the DSSP software for the eight REST simulations. Each color represents a different secondary structure as explained in the legend at the bottom. Monomers + CUR.

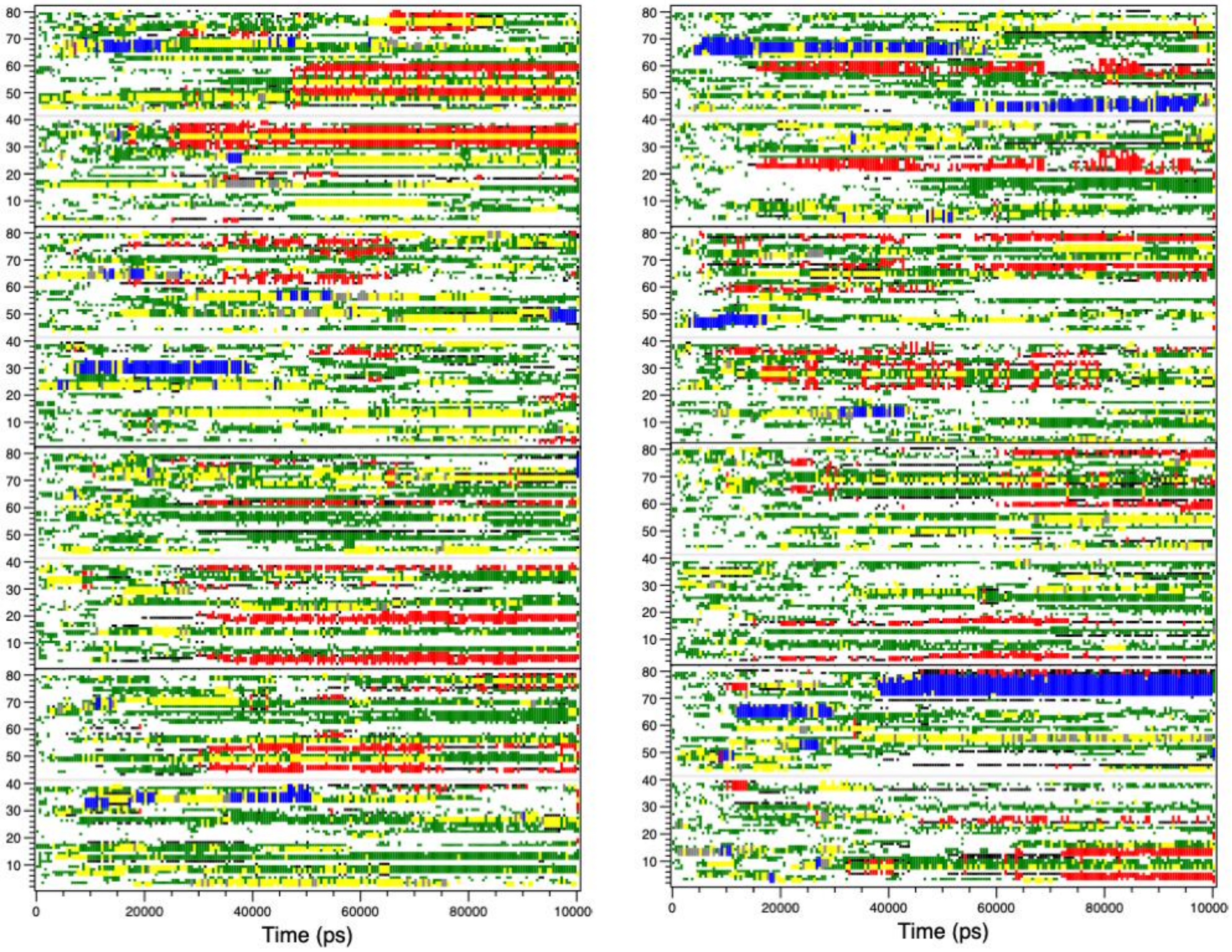

Figure S 5: Secondary structure assignment of two monomers for each residue obtained using the DSSP software for the eight REST simulations. Each color represents a different secondary structure as explained in the legend at the bottom. Monomers + CUR

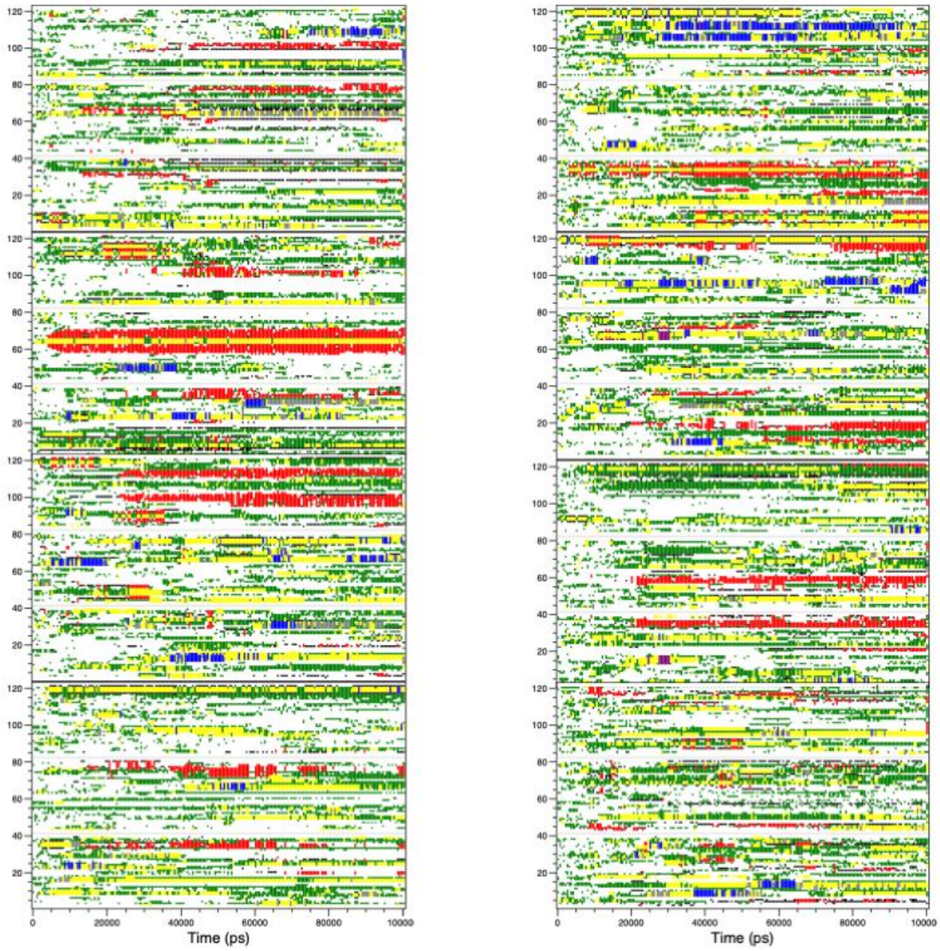

Figure S 6: Secondary structure assignment of three monomers for each residue obtained using the DSSP software for the eight REST simulations. Each color represents a different secondary structure as explained in the legend at the bottom. Monomers + CUR

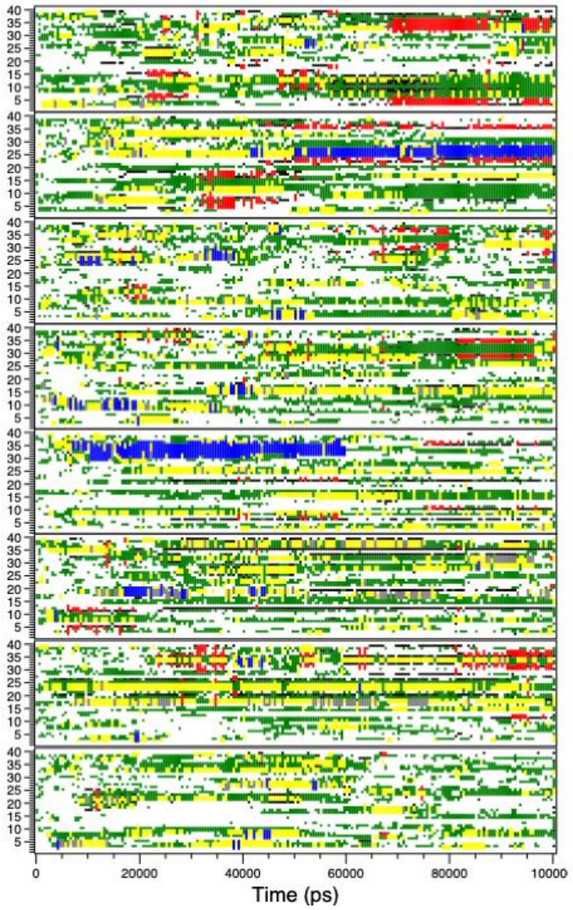

Figure S 7: Secondary structure assignment of the single monomer for each residue obtained using the DSSP software for the eight REST simulations. Each color represents a different secondary structure as explained in the legend at the bottom. Monomers + EGCG

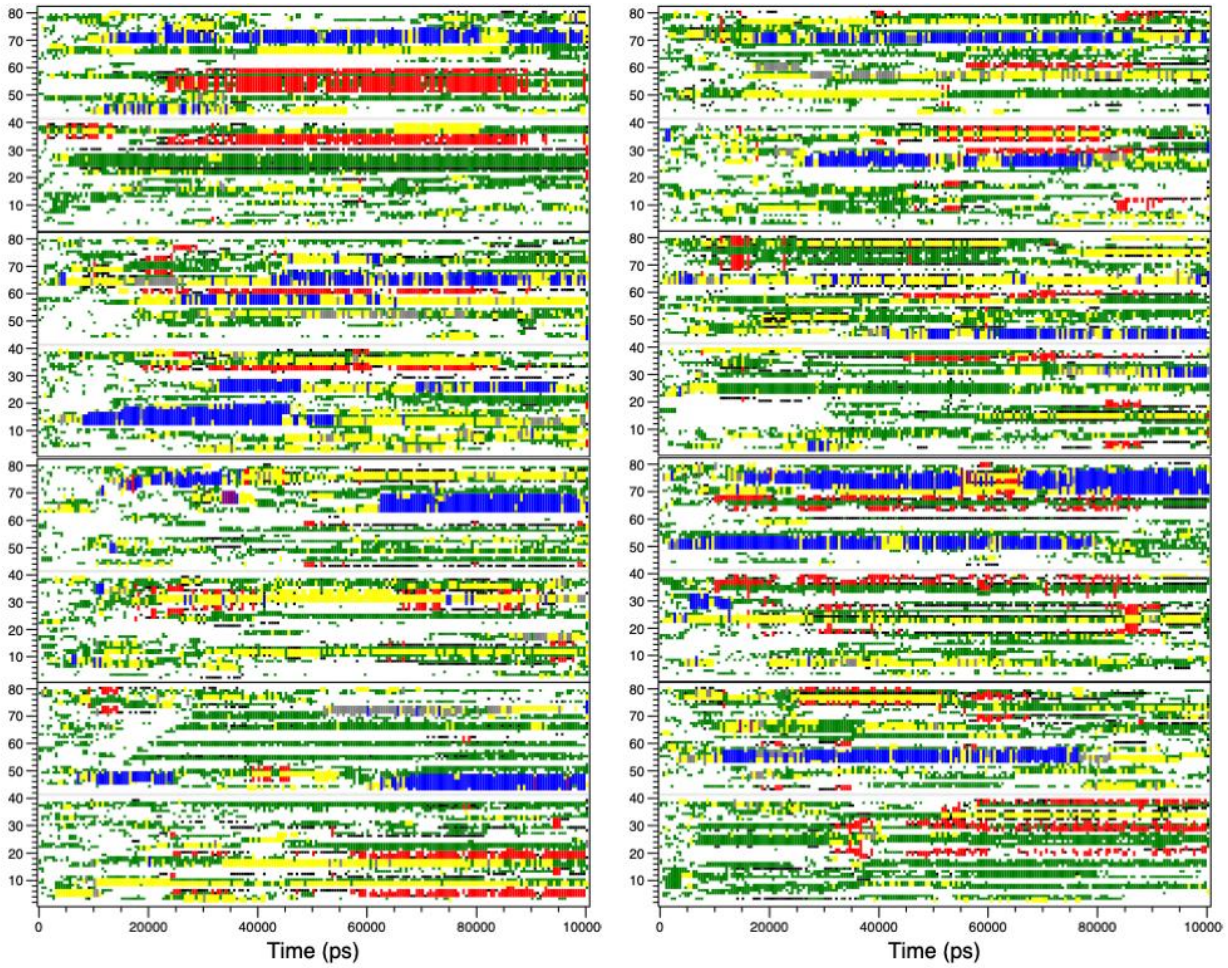

Figure S 8: Secondary structure assignment of two monomers for each residue obtained using the DSSP software for the eight REST simulations. Each color represents a different secondary structure as explained in the legend at the bottom. Monomers + EGCG

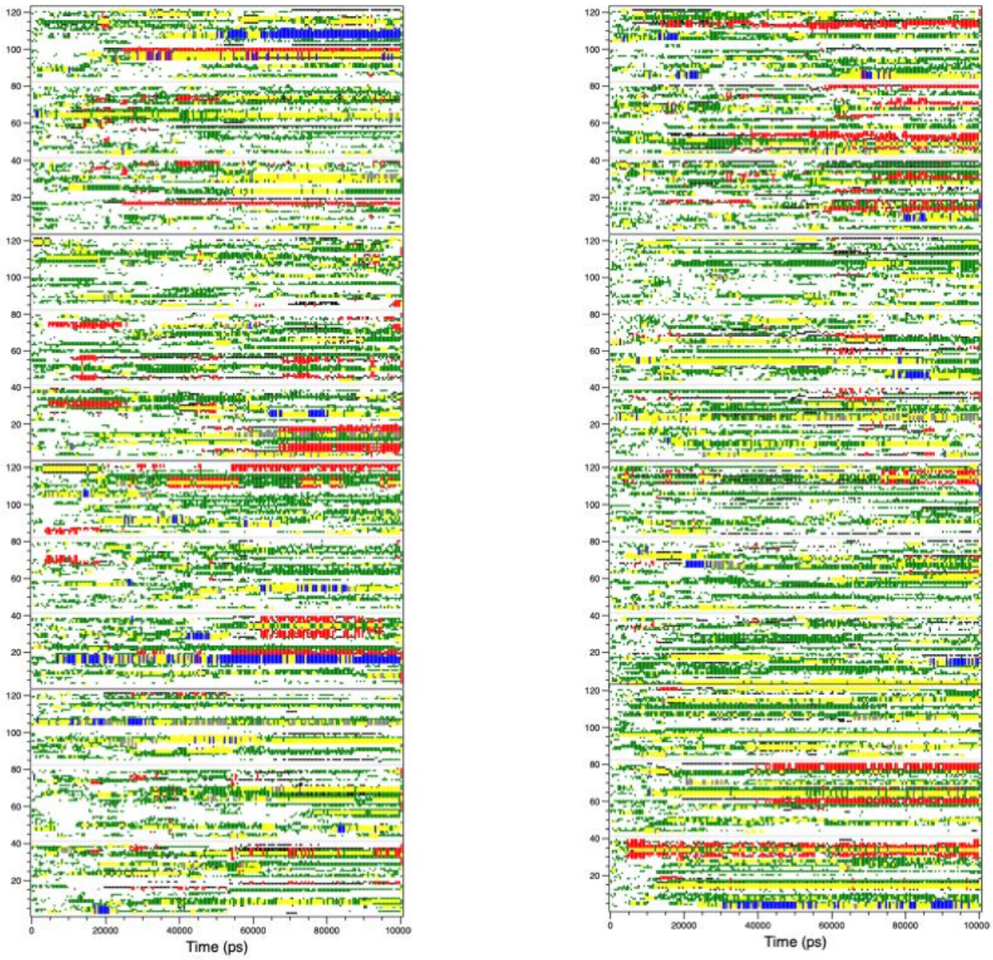

Figure S 9: Secondary structure assignment of three monomers for each residue obtained using the DSSP software for the eight REST simulations. Each color represents a different secondary structure as explained in the legend at the bottom. Monomers + EGCG
